# Antitumor and antimetastatic activities of chloroform extract of medicinal mushroom *Cordyceps taii* in mouse models

**DOI:** 10.1186/s12906-015-0762-9

**Published:** 2015-07-09

**Authors:** Ru-Ming Liu, Xiao-Jie Zhang, Gui-You Liang, Yong-Fu Yang, Jian-Jiang Zhong, Jian-Hui Xiao

**Affiliations:** Guizhou Center for Translational Medicine & Laboratory of Cell Engineering, Affiliated Hospital of Zunyi Medical University, Zunyi, 563000 China; Department of Pathology, Affiliated Hospital of Zunyi Medical University, Zunyi, 563000 China; State Key Laboratory of Microbial Metabolism, Joint International Research Laboratory of Metabolic & Developmental Sciences, and School of Life Sciences & Biotechnology, Shanghai Jiao Tong University, Shanghai, 200240 China

**Keywords:** Chinese traditional medicine, *Cordyceps taii*, Anticancer active ingredients, Antimetastatic activity, Tumor-burdened mouse model

## Abstract

**Background:**

*Cordyceps taii*, an entomogenous fungus native to south China, is a folk medicine with varieties of pharmacological activities including anticancer effect. To validate the ethnopharmacological claim against cancer, the antitumor and antimetastatic activities of chloroform extract of *C. taii* (CFCT) were investigated *in vivo*.

**Methods:**

The *in vitro* cytotoxic activities of CFCT against human lung cancer (A549) and gastric cancer (SGC-7901) cells were evaluated using the Sulforhodamine B (SRB) assay. *In vivo* anti tumor and antimetastatic activities, Kunming mice bearing sarcoma 180 and C57BL/6 mice bearing melanoma B16F10 were employed, respectively. The antitumor effects of CFCT were completely evaluated on the basis of the tumor weight, survival time, histologic analysis, and immune organ indices. The histopathological change, metastatic foci and malignant melanoma specific marker HMB45 in the lung tissue were detected for the evaluation of the antimetastatic activity of CFCT.

**Results:**

CFCT exhibited dose- and time-dependent cytotoxicities against A549 and SGC-7901 cells with the IC_50_ values of 30.2 and 65.7 μg/mL, respectively. Furthermore, CFCT at a dose of 50 or 100 mg/kg could significantly inhibit the tumor growth *in vivo* and prolonged the survival time in two different models as compared with the model group, especially when combined with the CTX at a low dose rate. And it also increased spleen index of Kunming mice and thymus index of C57BL/6 mice. Meanwhile, histologic analysis illustrated that CFCT alone or in combination with CTX could induce tumor tissue necrosis of both models. In addition, CFCT at a dose of 50 or 100 mg/kg inhibited the lung metastasis of melanoma B16F10 in tumor-bearing C57BL/6 mice. The antimetastatic effect was also observed when CFCT was used in combination with CTX. In comparison to any other groups, CFCT at a dose of 100 mg/kg could effectively enhance the GSH-Px activities of various tissues in tumor-bearing C57BL/6 mice.

**Conclusions:**

These findings demonstrate that CFCT has potent *in vivo* antitumor and antimetastatic activities, and may be helpful to the development of anticancer chemopreventive agents from *C. taii*.

## Background

Due to substantial morbidity and high mortality, cancer is considered as the second “killer” in the world [1]. A latest global cancer trend report by the World Health Organization shows that new cancer cases worldwide will remain a rapid increase, and reach more than 19 million a year by 2025 [2]. Cancer is a major global health crisis accordingly, and urgently needs effective prevention measures to curb the disease. Although the development of biological therapies have been used as new strategies for cancer treatment in recent years, surgery, radiotherapy and chemotherapy are still the three most frequently used therapies in the world. In comparison with surgery and radiotherapy, chemotherapy possess some advantages like systemic treatment and not causing significant physical damage, and it is recommended for patients with extrapelvic metastases or recurrent disease who are not candidates for the other two treatments [3]. However, long-term use of single-agent or combination chemotherapy usually results in severe side effects and resistance [4]. Obviously there is an urgent demand for exploring new chemotherapy drugs with high efficiency and low toxicity. The discovery of taxol and camptothecin lights up the hope for searching new anticancer drugs from natural sources.

The genus *Cordyceps*, an entomogenous fungus with a wide variety of pharmacological properties, is a well-known and valuable source of traditional Chinese medicine. The applications of *Cordyceps* in China and other Eastern Asian countries suggest that it may be used for cancer prevention and treatment [5]. Furthermore, current *Cordyceps* has received considerable attention worldwide as a potential source of anticancer drugs [6, 7]. *Cordyceps taii* is a folk medicine native to south China [8]. Previously, polysaccharides from *C. taii* were found to display immunomodulatory, antitumor, and antimutagenic activities [8]. Our recent findings suggest that *C. taii* polysaccharides are a promising source of natural antioxidant and antiaging agents [9]. Further pharmacological experiments indicate that *C. taii* has broad-spectrum antimicrobial effects including potent antibacterial and antifungal activities, and the active ingredients were found to be enriched in non-polar fractions extracted with organic solvents such as chloroform, ethyl acetate, and acetone [10, 11]. However, the anticancer potential of these non-polar fractions of *C. taii* has never been investigated. In view of the broad spectrum of therapeutic potentials of *Cordyceps* spp., the aim of the present study was to assess the antitumor and antimetastatic activities of chloroform extract of *C. taii* (CFCT) *in vivo*, and to promote the further development of anticancer active ingredients from *C. taii*.

## Methods

### Chemicals and reagents

Dimethylsulfoxide (DMSO), sulforhodamine B (SRB), Tris, trypsin, and trypan blue were purchased from Sigma-Aldrich (USA). The RPMI-1640 medium, 4-(2-hydroxyethyl)-1-piperazineethanesulfonic acid (HEPES), *L*-glutamine, and fetal calf serum were purchased from Gibco (USA). Cyclophosphamide (CTX) was purchased from Hengrui Pharmaceuticals, Co. Ltd. (Nanjing, China). Olive oil was purchased from Zhongqi Huaye Inc. (Beijing, China). Histo-clear agent was purchased from ESM Inc. (USA). HMB45 anti-melanoma antibody was purchased from Abcam, PV-6002 non-biotin two-step IHC detection kit (Horseradish peroxidase-conjugated goat antimouse IgG secondary antibody) was supplied by Zhongshan Golden Bridge Biotechnol. Co. Ltd. (Beijing, China), DAB Horseradish Peroxidase Color Development Kit was purchased from Beyotime Ins. Biotechnol (Haimen, China). Glutathione peroxidase (GSH-Px) antioxidative enzyme detection kit was from Jiancheng Bioengin Ins (Nanjing, China). All chemicals used were of analytic grade.

### Medicinal fungus, cultivation, and mycelia preparation

The voucher specimens of *C. taii* (strain GYYA 0601) were deposited at the Laboratory of Microbial Resources & Drug Development, Center for Translational Medicine, Affiliated Hospital of Zunyi Medical University, Guizhou Province, China. The mycelia of *C. taii* were cultured and harvested as previously described [12, 13]. Subsequently, the mycelia were lyophilized and grinded (60 mesh to 100 mesh) for later experiments.

### Preparation of active fraction

The dried mycelia power of *C. taii* (4400 g) was extracted five times with 25 L of 80 % (v/v) aqueous-methanol solution by hot soaking. The collected extract solutions were filtered through a 0.45 μm pore-size filter, and their solvents were then removed at 40 °C by a rotary evaporator under vacumm to yield a brown MeOH extract (2252 g). Subsequently, the extract was resuspended in hot water. The suspension was extracted five times with equal volumes of petroleum ether (b.p.60–90°) to yield the petroleum ether extract, and the aqueous residue was further extracted five times with equal volumes of chloroform. Finally, the combined chloroform layers were evaporated using a rotary evaporator under reduced pressure to yield the chloroform extract of *C. taii* (CFCT, 210 g). Stock solution of CFCT was prepared in DMSO and stored at −20 °C. Further dilution was made with medium just before use, and the final concentration of DMSO was less than 0.1 % (v/v).

### Cell line and culture

The human lung cancer cell line A549, gastric cancer cell line SGC-7901, and the mice cancer cell lines such as sarcoma 180 and melanoma B16F10 were purchased from the Cell Bank of Type Culture Collection of the Chinese Academy of Sciences (Shanghai, China). All the above four cell lines were grown in RPMI 1640 medium supplemented with 10 % fetal bovine serum, 25 mM HEPES buffer, and 2 mM l-glutamine in a humidified incubator (Thermo, USA) preset to 37 °C and 5 % CO_2_. Cells at the logarithmic growth stage were used for all experiments.

### Animals

Kunming (KM) mice and C57BL/6 mice (male; age: 5 to 7 weeks; weight: 18.0 ± 2.0 g) were provided by the Experimental Animal Center of the Third Military Medical University in Chongqing, China (Animal License No. SCXK (YU) 2007–2006). The animals were kept in a standard laboratory environment and fed with sterile pellets and water *ad libitum*. The laboratory animal protocol for this study was approved by the Zunyi Medical College Committee for the Control and Supervision of Experimental Animals. All experimental animals were bred for 7 d before use.

### Assay of cytotoxic activity

The SRB colorimetric method was employed, as previously described, to measure the cytotoxic activities of CFCT against human cancer cells, including the dose- and time-responses [14]. CFCT was dissolved in DMSO and stored at −20 °C. The thawed samples were immediately diluted in RPMI 1640 medium to reach a concentration of 1 mg/mL before further use. For the analysis of the dose–response, cancer cells were seeded into 96-well plates at a density of 6 × 10^3^ cells in 100 μL complete medium per well and were incubated at 37 °C for 24 h. Eight concentrations of CFCT (100 μL) were added to each well, and the final concentrations were 1.9, 3.9, 7.8, 15.6, 31.25, 62.5, 125.0, and 250.0 μg/mL, respectively. These samples were further incubated for 48 h. A blank control group (culture media only), a negative control group (cells cultured in the media), a positive control group (cells were treated with 25 μg/mL DDP), and a solvent control group (cells treated with DMSO at the same concentration as the treatment drug group) were all placed in the same 96-well plate. After 48 h of drug exposure, its cytotoxic effect was detected using a SRB colorimetric method as previously reported [14]. The absorbance data were exported into a Microsoft Excel spreadsheet (Microsoft) for further analysis. Cell densities were obtained by correcting the absorbance based on the blank controls. Cell survival and/or cell growth inhibitory rate was calculated as the percentage absorbance compared with that of the negative control. Likewise, cancer cells were exposed to 40 μg/mL of CFCT for 8, 20, 32, 44, 56, 68 and 76 h, respectively, for analysis of the time response.

### Antitumor activity of CFCT in subcutaneous sarcoma 180-bearing mice

The sarcoma-180 (S180) solid tumor model was empolyed to assess the antitumor effect of the CFCT *in vivo* on the basis of the tumor growth and host survival. Tumor cells were harvested from the peritoneal cavity of KM mice with a 10-day-old S180 ascitic tumor under the sterile condition, washed twice with sterile saline, and suspended in the sterile saline at a density of 1 × 10^7^ cells/mL. KM mice were then inoculated subcutaneously (s.c.) 0.2 mL of tumor cells suspension per mouse into their right hind limbs on day 0. Subsequently, the mice were randomly divided into seven groups (n = 16). CFCT was dissolved in a certain amount of olive oil. The CFCT treatment groups were administered i.p. daily to the mice at doses of 20, 50 and 100 mg/kg (LG, MG and HG groups) for 7 consecutive days, starting 24 h after tumor transplantation. A combined administration group (CG group, 20 mg/kg of CFCT + 20 mg/kg of CTX, once daily), a positive control group (PG group, 20 mg/kg of CTX, once daily), and a negative control group (SG group, mice were treated with olive oil at the same volume as the CFCT treatment group) were also employed on the same schedule. The Model group was treated with saline only. The tumor size was measured with digital calipers every day, and its volume (cm^3^) was calculated as the (length × width^2^)/2. Two independent experiments were performed for each treatment with eight mice per group. On day 8, eight mice from each group were anaesthetized and sacrificed by cervical dislocation, and their bodies were weighed. Simultaneously, their solid tumor, thymus and spleen were quickly removed and weighted, respectively. The tumors were fixed in 10 % paraformaldehyde for at least 24 h, and then were embedded in paraffin under vaccum. While the rest were allowed to live to a natural death, and the death time was recorded to calculate the median survival time (MST). The percentage increase in life span (ILS) of tumor hosts was calculated on the basis of mortality of the experimental mice: MST = ΣSurvival time of each mouse in a group/Total number of mice; %ILS = (MST of treated group/MST of control group) × 100. The immune organ indices were defined as the thymus and/or spleen weight relative to body weight. Spleen (thymus) index = Spleen (thymus) weight/body weight × 100 %. The tumor inhibitory ratio = (the average tumor weight of model group - the average tumor weight of treatment group)/the average tumor weight of model group × 100 %.

### Antitumor and antimetastatic activities of CFCT in subcutaneous melanoma B16F10-bearing mice

Cultured murine melanoma B16F10 cells during the exponential phase of growth were harvested by trypsinization, washed, and suspended at 1.5 × 10^6^ cells/mL in RPMI-1640 medium supplemented with 10 % FBS. C57BL/6 mice (n = 112) were injected s.c. with 2.25 × 10^5^ B16F10 cells per mouse into the lower right groin on day 0, and were then randomly assigned into seven groups (n = 16). After the implantation of tumor, 0.2 mL CFCT (20, 50 and 100 mg/kg) was administered i.p. once every other day for two weeks in the treatment group. As described above, two independent experiments were performed for each treatment with eight mice per group, and the CG, PG, SG, and Model groups were also given on the same schedule. On day 15, eight mice from each group were anaesthetized and sacrificed by picking off the eyeballs, and their blood sample, liver, brain were quickly harvested. All tumor, thymus, spleen and lung were also removed and weighted for the assession of antitumor and antimetastic activities and side-effects. Invasive metastases to the lung were observed manually. The rest of mice were used as assessing the survival time.

### Histological examination and immunohistochemistry staining

For histopathology studies, all tumors were washed by normal saline, fixed by 10 % paraformaldehyde in phosphate buffer saline, successively dehydrated in solutions containing an increasing percentage of ethyl alcohol (70, 80, 95 and 100 %), embedded in paraffin under a vacuum, cut into 5 μm-thick sections, deparaffinized in histo-clear agent, and stained with Harris hematoxylin-eosin (HE staining).

For immunohistochemical staining, lung tissues in C57BL/6 mouse were fixed for at least 24 h by Bouin soultion. Lung tissue sections of 5 μm-thick were dried overnight at 65 °C and deparaffinized in histo-clear. The sections were rehydrated through graded alcohols into water. After rehydrating, antigen retrieval was carried out by heating for 20 min at 100 °C in 10 mM citrate buffer (pH 6.0). Endogenous peroxidase activity was blocked with 3 % H_2_O_2_ in methanol for 10 min at room temperature and non-specific binding of reagents was quenched by 10 % normal goat or rabbit serum. After rinsing with distilled water for 5 min, the sections were incubated at 4 °C to stay overnight with primary anti-mouse HMB45 monoclonal antibody. The sections were rinsed with PBS again for 2 min, and then were incubated at 37 °C for 30 min with horseradish peroxidase-conjugated goat anti-mouse IgG secondary antibody. After washing by PBS, the sections were stained using a DAB kit and observed under microscope. Appropriate positive and negative controls without primary antibody were included.

### Analysis of the antioxidant endogenous enzyme GSH-Px

The defense effect of CFCT on antioxidant-related endogenous enzyme GSH-Px was investigated. Blood samples were collected from the orbital venous plexuses of the mice under anesthesia. The brains and livers were rapidly excised and thoroughly washed to clear off blood. These organs were immediately transferred to ice-cold saline and homogenized (10 %) in cold saline (about 4 °C). The blood and homogenate tissues were centrifuged at 3000 × g and 4 °C for 20 min. GSH-Px in the supernatants was assessed using the respective detection kits as previously described [9].

### Statistical analysis

For each measured drug concentration, there were five to eight identical wells in the 96-well culture plates. All experiments were performed at least three times. The experimental data were statistically analyzed using the SPSS (version 13.0) software, and the data were expressed as the means with their corresponding standard errors. When appropriate, statistical significance was analyzed using a two-tailed Student’s *t*-test. Differences were considered statistically significant if *P* < 0.05.

## Results

### Cytotoxic activity of CFCT against human cancer cells *in vitro*

**Fig. 1:**
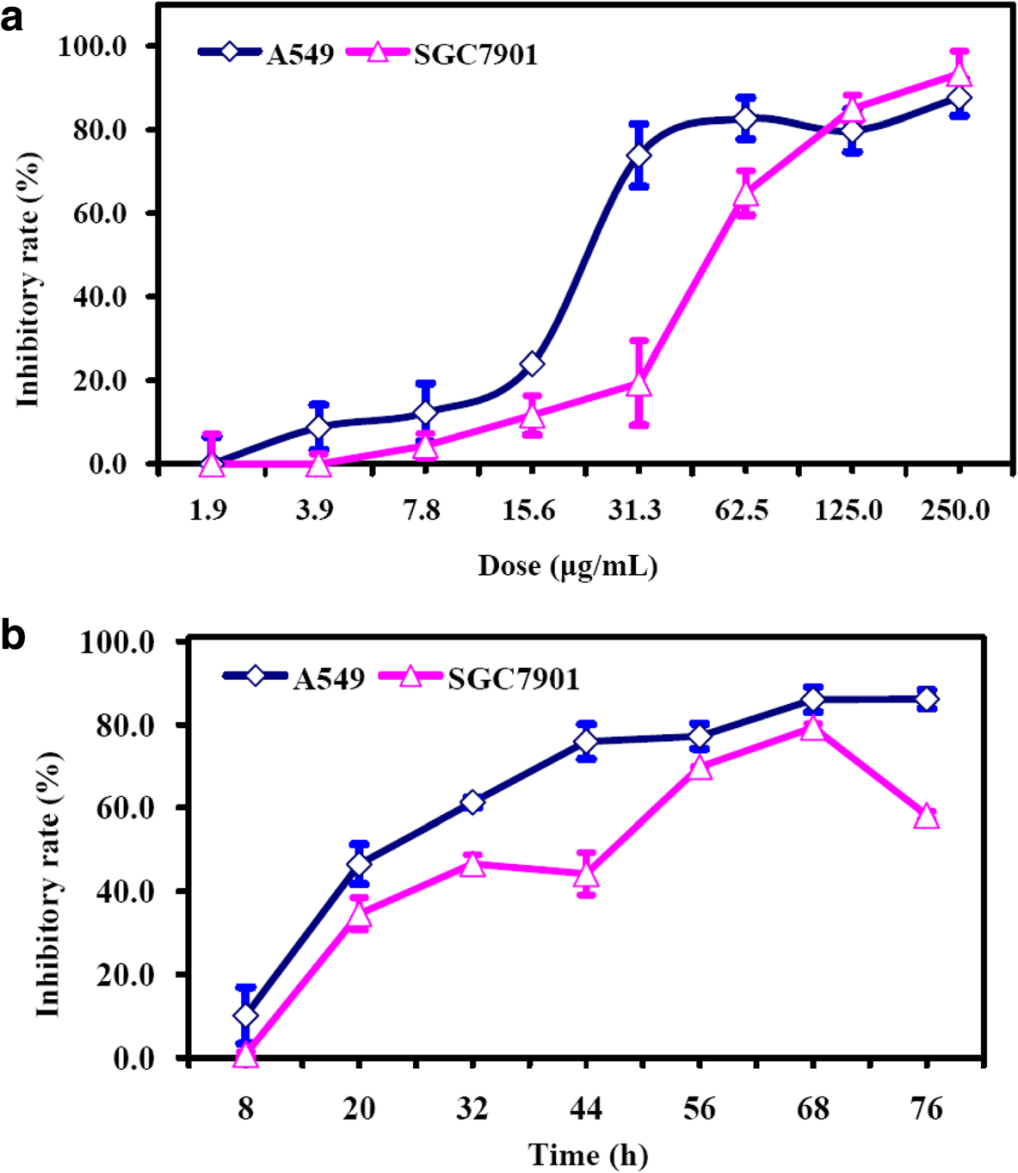
Cytotoxicity of CFCT against human lung carcinoma A549 cells and human gastric carcinoma SGC-7901 cells. **a** A549 cells and SGC-7901 cells treated with different concentrations of CFCT for 48 h; **b** A549 cells and SGC-7901 cells treated with CFCT (40 μg/mL) for a different period (8–76 h). Data were shown as mean ± SD (n = 3)

The cytotoxic activities of CFCT against two different cancer cell lines, *i.e.* human lung carcinoma A549 cells and human gastric carcinoma SGC-7901 cells, were displayed in Fig. 1. CFCT exerted potent cytotoxic activities in a dose-dependent manner at the dose range from 1.9 to 250.0 μg/mL after 48 h of exposure (Fig. 1a). The IC_50_ values of CFCT against cancer cells were calculated to be 30.2 ± 2.6 and 65.7 ± 5.3 μg/mL for A549 and SGC-7901 cells, respectively. Therefore, the A549 cells showed about two-fold sensitivity toward CFCT in comparision to SGC-7901 cells. As shown in Fig. 1b, the cytotoxic activities of CFCT against A549 cancer cells presented approximately in a time-dependent manner within the time range tested at a dosage of 40 μg/mL, but a non-time-dependent manner for SGC-7901 cells.

### Antitumor effect of CFCT in S180 tumor-bearing KM mice

**Fig. 2:**
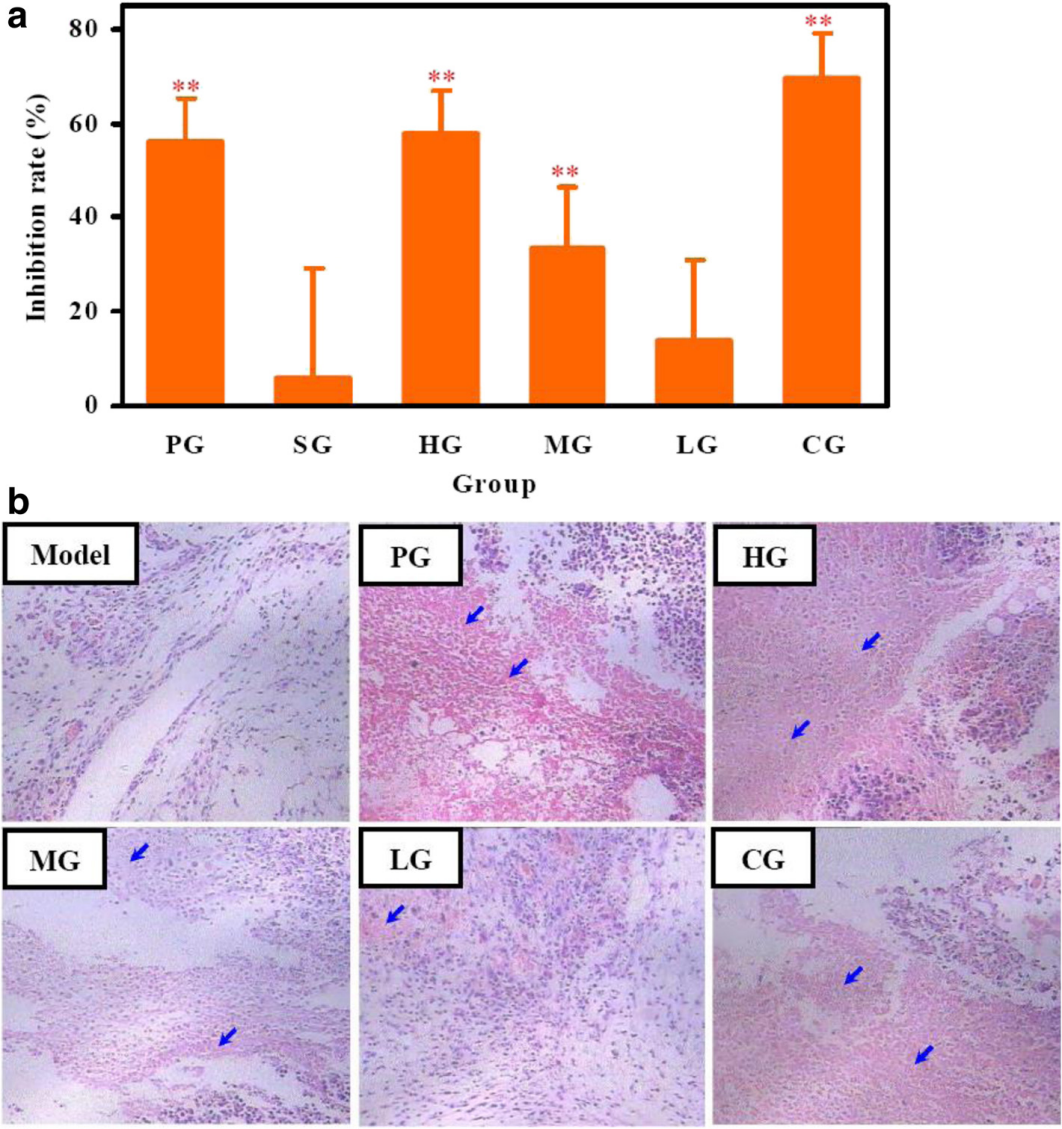
Antitumor effect of CFCT in S180 tumor-bearing KM mice. **a** Inhibitory rate in treatment groups. Values were expressed as mean ± SD (n = 8). The inhibitory ratio = (the average tumor weight of model group - the average tumor weight of treatment group)/the average tumor weight of model group × 100 %; **b** Representative HE staining sections from different groups. Blue arrows denote necrotic tissues. **P < 0.01 *vs.* model group. Model group, mice were treated with olive oil at the same volume as the CFCT treatment group; PG, mice were treated with cytoxan (20 mg/kg); SG, mice were treated with saline at the same volume as the CFCT treatment group; HG, mice were treated with CFCT (100 mg/kg); MG, mice were treated with CFCT (50 mg/kg); LG, mice were treated with CFCT (20 mg/kg); CG, mice were treated with cytoxan (20 mg/kg) and CFCT (20 mg/kg)

As shown in Table 1, the tumor weight of HG, MG, and CG groups was decreased significantly compared with that of the model group. However, no significant difference of tumor weight was observed between LG and model groups. The inhibitory ratio of HG, MG and CG groups was 57.9 ± 8.9 %, 33.6 ± 12.8 %, and 69.4 ± 9.7 % (Fig. 2a), respectively, which presented significant differences in comparison with model control (*P* < 0.01). Furthermore, the inhibitory ratio of the positive control (56.1 ± 9.1 %) was also lower than both HG and CG groups. Further histopathological analysis of tumor tissue by HE staining showed a large number of necrotic cancer cells or tissues (blue arrow denote necrotic cells) in the HG, MG and CG groups (Fig. 2b). Accordingly, these data indicated that CFCT could significantly inhibit tumor growth in S180 tumor-bearing KM mice.

**Table 1:** Effects of CFCT on tumor weight and immune organ indices on day 8 in S180 tumor-bearing KM mice

Group	Dosage (mg/kg)	Tumor weight (g)	Spleen index (mg/g)	Thymus index (mg/g)
Model	─	0.97 ± 0.19	4.683 ± 1.149	2.994 ± 0.702
PG	20.0	0.42 ± 0.09**	5.600 ± 0.667	3.060 ± 0.397
SG	─	0.91 ± 0.23	4.484 ± 1.064	2.611 ± 0.375
HG	100.0	0.41 ± 0.09**	6.288 ± 0.983*	2.512 ± 0.190*
MG	50.0	0.64 ± 0.12**	5.750 ± 0.907*	2.230 ± 0.293**
LG	20.0	0.83 ± 0.26	4.864 ± 0.584	2.587 ± 0.230*
CG	(LG + PG)	0.30 ± 0.09**	5.751 ± 1.146*	2.723 ± 0.410

Values are mean ± SD (n = 8). **P < 0.01, *P < 0.05 *vs.* model control group. All groups as described with Fig. 2

### Antitumor effect of CFCT in melanoma B16F10-bearing C57BL/6 mice

**Fig. 3:**
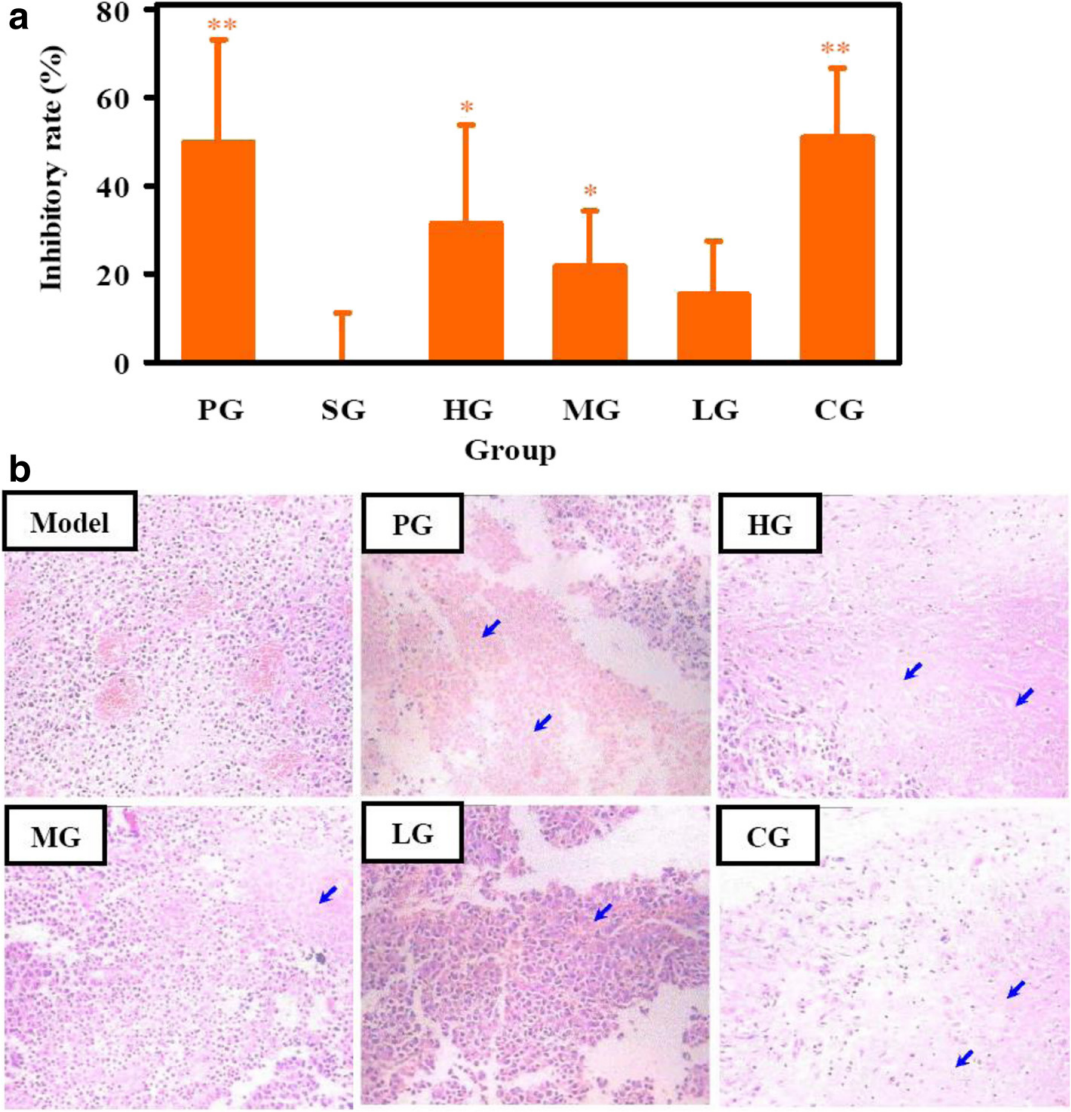
Antitumor effect of CFCT in melanoma B16F10-bearing C57BL/6 mice. **a** Inhibitory rate in treatment groups. Values were expressed as mean ± SD (n = 8). The inhibitory ratio = (the average tumor weight of model group - the average tumor weight of treatment group)/the average tumor weight of model group × 100 %; **b** Representative HE staining sections from different groups. Blue arrows denote necrotic tissues. *P < 0.05, **P < 0.01 *vs.* model group. All groups as described with Fig. 2

CFCT could inhibit the tumor growth of murine metastatic melanoma B16F10-bearing mice in comparison with the model group, especially in the HG and CG groups (Table 2). As shown in Fig. 3a, it resulted in more than 30 % inhibition in both HG group (*P* < 0.05) and CG group (*P* < 0.01) compared with the model group. However, no significant difference of tumor weight was observed between LG group and the model group. Further histopathological analysis of tumor tissue with HE staining displayed a large number of necrotic cancer cells or tissues (blue arrow denote necrotic cells) in the HG and CG groups (Fig. 3b). Therefore, these data suggested that CFCT could moderately inhibit tumor growth in B16F10-bearing C57BL/6 mice.

**Table 2:** Effects of CFCT on tumor weight and immune organ indices on day 15 in melanoma B16F10-bearing C57BL/6 mice

Group	Dosage (mg/kg)	Tumor weight (g)	Spleen index (mg/g)	Thymus index (mg/g)
Model	─	2.07 ± 0.29	9.648 ± 1.992	1.133 ± 0.288
PG	20.0	1.04 ± 0.48**	8.067 ± 1.300	1.955 ± 0.460**
SG	─	2.12 ± 0.31	9.152 ± 1.783	0.954 ± 0.291
HG	100.0	1.42 ± 0.47*	6.858 ± 1.321*	1.800 ± 0.521*
MG	50.0	1.62 ± 0.26*	7.180 ± 1.437*	1.603 ± 0.427*
LG	20.0	1.76 ± 0.25	7.080 ± 2.369*	1.164 ± 0.390
CG	(LG + PG)	1.01 ± 0.32**	8.284 ± 1.806	1.136 ± 0.466

Values are mean ± SD (n = 8). **P < 0.01, *P < 0.05 *vs.* model control group. All groups as described with Fig. 2

### Immune organ index and survival prolongation of tumor-bearing mice after CFCT treatment

Thymus and spleen are the primary immune organs and directly affect the organism’s immune function [15]. The effects of CFCT on the thymus index and spleen index of both tumor-bearing mouse models were shown in Table 1 and Table 2. Compared to the model control group, the spleen index of KM mice and the thymus index of C57BL/6 mice in the MG and HG groups were significantly increased (*P* < 0.05). However, CFCT at all dosages tested had obvious inhibitory effects on the thymus index of KM mice and the spleen index of C57BL/6 mice (*P* < 0.05).

**Fig. 4:**
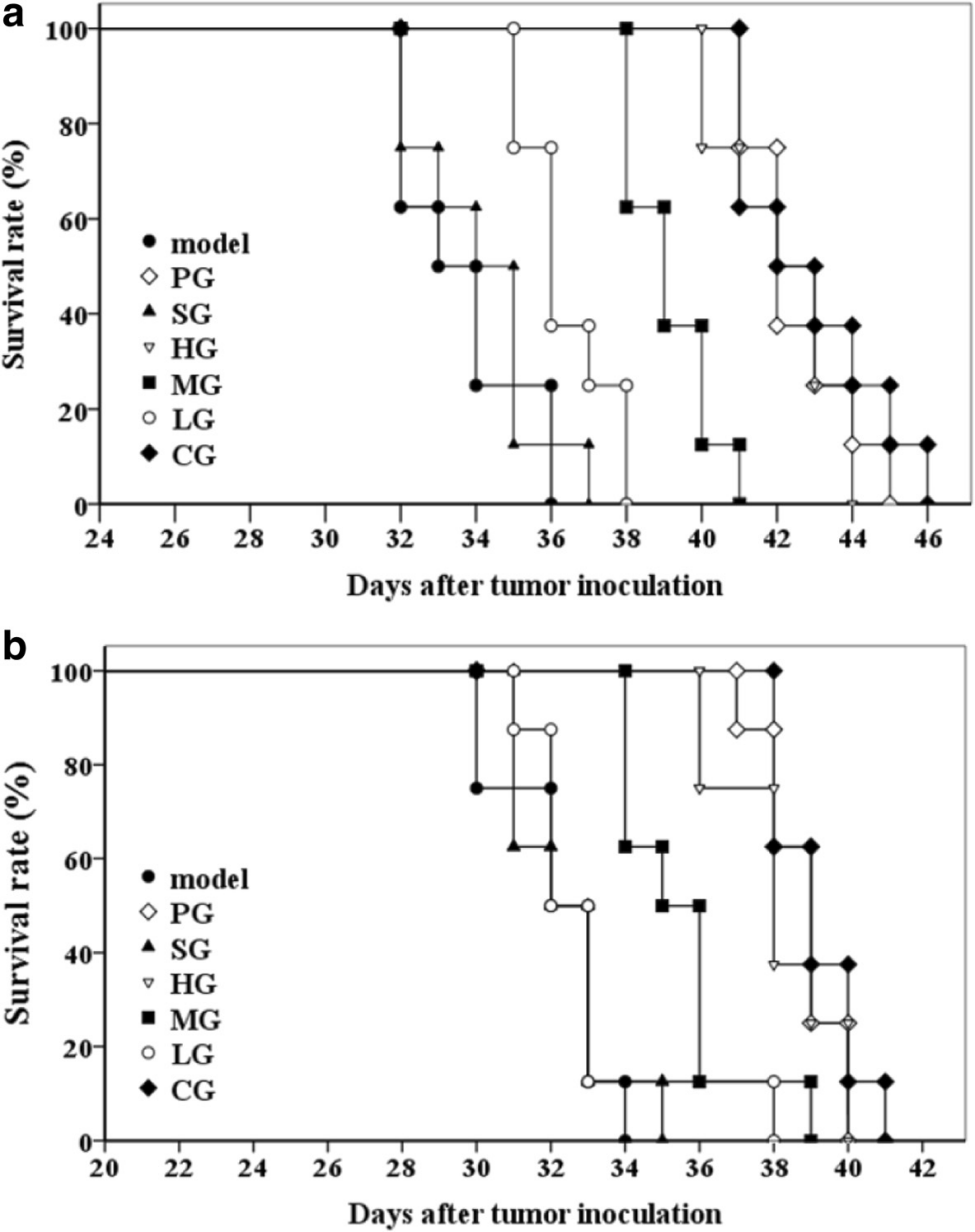
Survival prolongation of tumor-bearing models after CFCT treatment. **a** Survival time of S180 tumor-bearing KM mice; **b** Survival time of melanoma B16F10-bearing C57BL/6 mice. Graph shows Kaplan-Meier survival curves for tumor-bearing mice treated with different groups. Survival was significantly longer in LG-treated mice *vs*. model group (log-rank test, *P* < 0.05). Data were presented as mean ± SD (n = 8). All groups were the same as described in the legends of Fig. 2

The beneficial effects of CFCT on tumor-bearing mouse models were also reflected in the survival time. Survival times of the tumor-bearing mice in the MG, HG and CG groups were significantly prolonged for both tumor-bearing models compared with the model control group (Table 3, Table 4, and Fig. 4, *P* < 0.01). The extension rate of lifespan of HG group was slightly lower than that of positive control group PG, but the extension rate of lifespan of CG group was higher than that of PG group (Table 3, Table 4, and Fig. 4).

**Table 3:** Effect of CFCT on survival rate in S180 tumor-bearing KM mice

Group	Dosage (mg/kg)	Survival time (d)	Survival prolongation rate (%)
Model	─	33.2 ± 1.7	─
PG	20.0	42.5 ± 1.4**	26.39 ± 0.82**
SG	─	34.1 ± 1.7	1.49 ± 0.11
HG	100.0	42.1 ± 1.6**	25.28 ± 0.14**
MG	50.0	39.1 ± 1.1**	16.36 ± 1.68**
LG	20.0	36.4 ± 1.2	8.18 ± 1.49
CG	(LG + PG)	42.9 ± 2.0**	27.51 ± 0.80**

Values are mean ± SD (n = 8). **P < 0.01 *vs.* model control group. All groups as described in Fig. 2

**Table 4:** Effect of CFCT on survival rate in B16-bearing C57BL/6 mice

Group	Dosage (mg/kg)	Survival time (d)	Survival prolongation rate (%)
Model	─	32.13 ± 1.46	─
PG	20.0	38.75 ± 1.04**	20.62 ± 1.32**
SG	─	32.38 ± 1.41	0.78 ± 0.16
HG	100.0	38.13 ± 1.55**	18.68 ± 0.30**
MG	50.0	35.50 ± 1.69**	10.51 ± 0.72**
LG	20.0	33.00 ± 2.14	2.72 ± 2.12
CG	(LG + PG)	39.13 ± 1.13**	21.79 ± 1.03**

Values are mean ± SD (n = 8). **P < 0.01 *vs.* model control group. All groups as described in Fig. 2

### Antimetastatic activity of CFCT in melanoma B16F10-bearing C57BL/6 mice

**Fig. 5:**
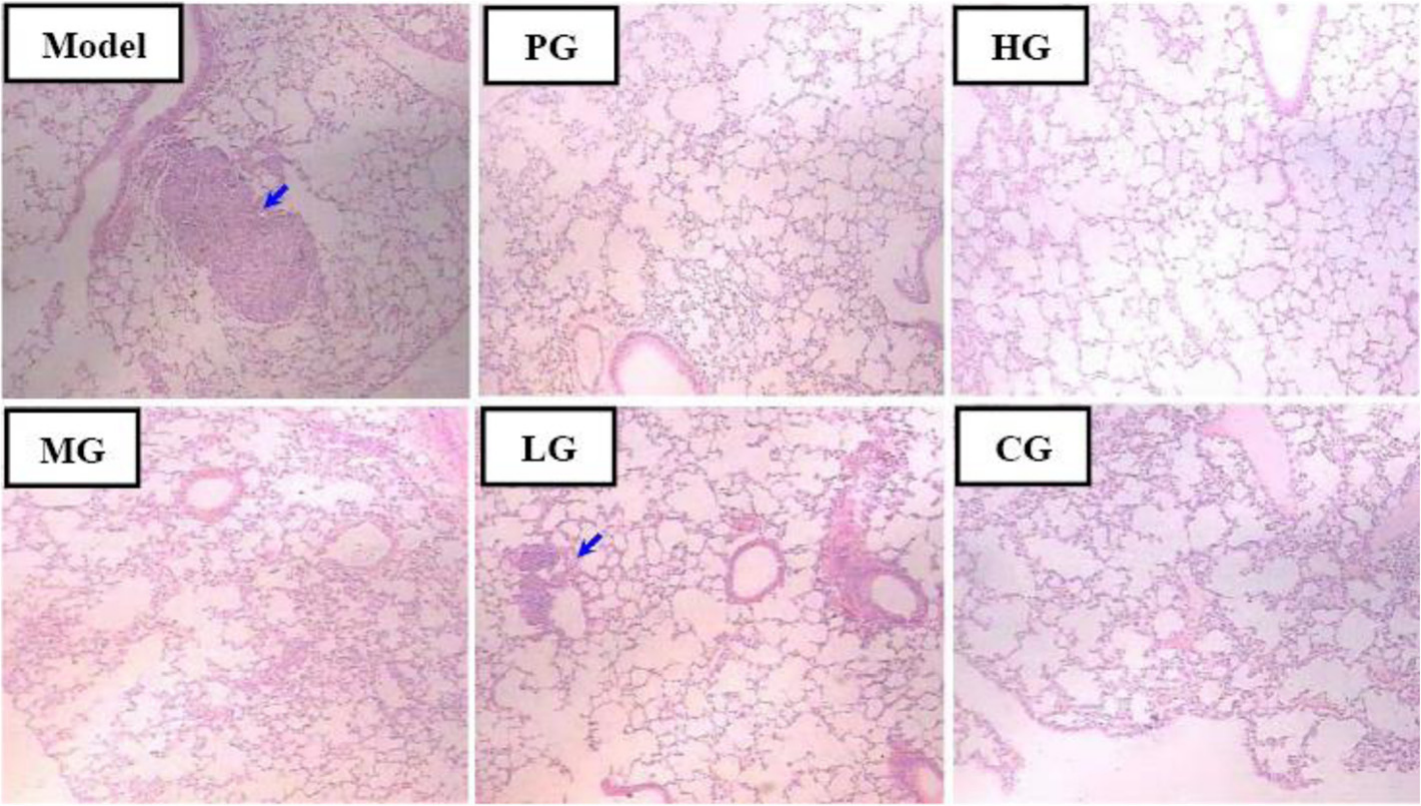
Pathological changes of lung tissue from B16F10-bearing C57BL/6 mice by HE staining after administration. Blue arrows denote metastasis. All groups as described in Fig. 2

Malignant melanoma, with high metastasis risk, often transfers to the lung tissue in C57BL/6 mice by blood metastasis, and is commonly used as a model of tumor metastasis [16]. Histopathological section of tumor-bearing C57BL/6 mice lung tissue with HE staining showed obviously metastatic foci of melanoma B16F10 (blue arrow) in the model and LG groups (Fig. 5), particularly in the model group. But metastatic foci were not observed in other groups.

**Fig. 6:**
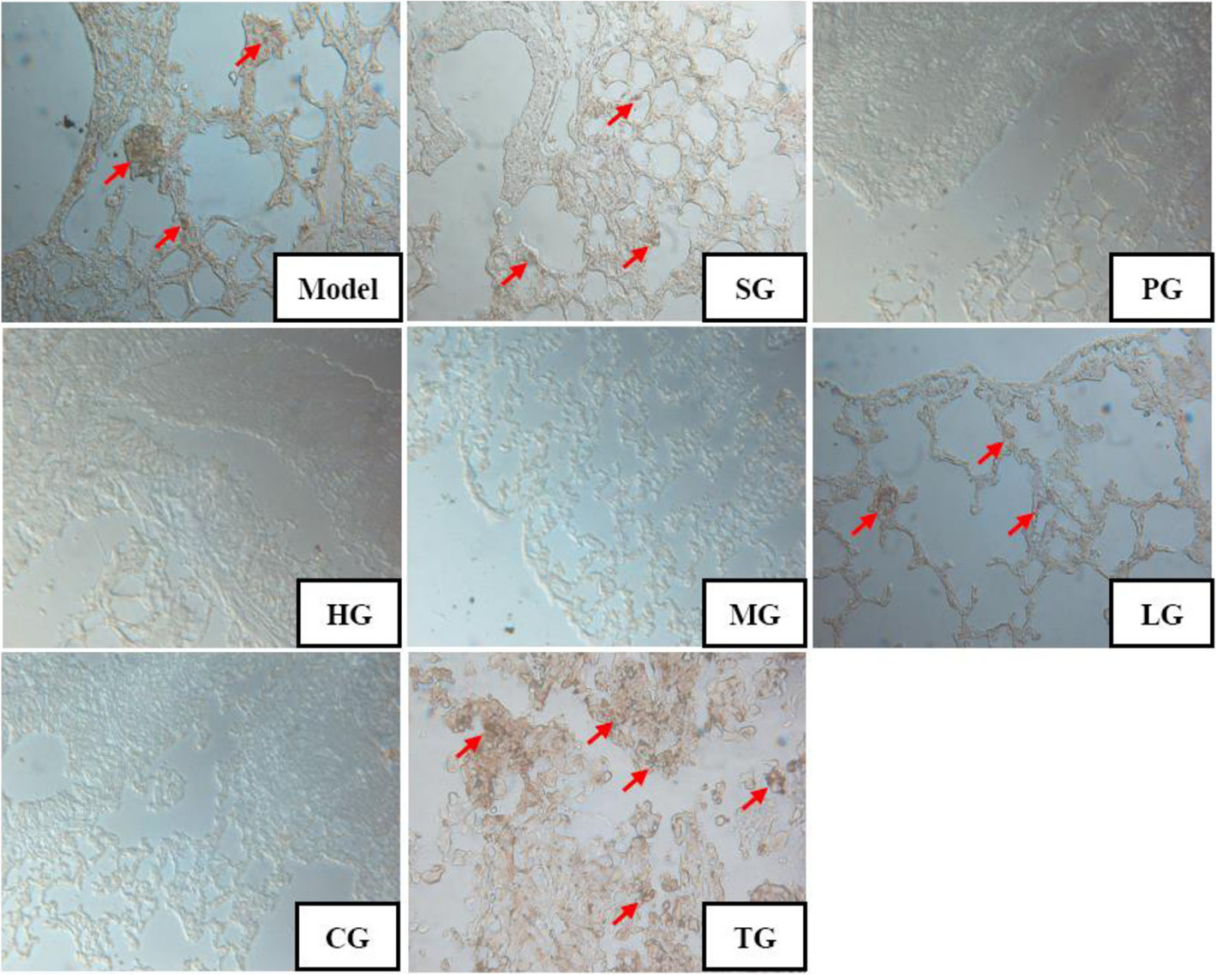
Effects of CFCT on immunohistochemistry in lung tissue from melanoma B16F10-bearing C57BL/6 mice. Light micrograph of HMB45-positive cells (original magnification × 400) stained by the anti-mouse HMB45 mouse monoclonal antibody. Red arrows denote HMB45 protein. TG: solid tumor tissue of model group. Other groups as described in Fig. 2

HMB45, molecular weight of 7000 kDa, is a specific protein marker for melanoma cells, and mainly distributed in the cytoplasm [17]. Further, the expression of HMB45 in the lung tissue of tumor-bearing C57BL/6 mice was assessed by the immunohistochemical staining method. As shown in Fig. 6, the cytoplasm was basically dyed clay-bank (red arrow), which indicated the aggregation of HMB45 positive cells in the solid tumor tissue (TG) of model group. Consistently, a clay-bank lump (red arrow), meaning small metastatic foci, was found in the lung tissue of model group, and it was different from the surrounding normal lung tissue. Only a few HMB45 positive cells were observed in the lung tissue of LG group, and they were not found in the MG, HG and CG groups. In addition, the HMB45 positive cells were more spread out in comparison to the model group and metastatic foci were not found in the lung tissue of solvent group (SG).

Based on the above results of HE and HMB45 immunohistochemistry staining, the HG and MG groups of CFCT could effectively inhibit the lung metastasis of malignant melanoma B16F10 in C57BL/6 mice.

### Effect of CFCT on the GSH-Px activity in melanoma B16F10-bearing C57BL/6 mice

**Fig. 7:**
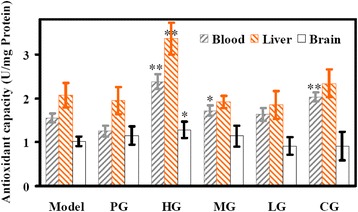
Effects of CFCT on antioxidant ability in different tissues of melanoma B16F10-bearing C57BL/6 mice. *P < 0.05, **P < 0.01 *vs.* model group. All groups as described in Fig. 2

Studies have shown that enhancement of antioxidant capacity is one of the main action mechanism of anticancer drugs [18]. In the present study, the effects of CFCT on the activities of endogenous antioxidant enzyme GSH-Px in the blood, brain, and liver tissues were investigated in melanoma B16F10-bearing C57BL/6 mice (Fig. 7). Compared with the model group, CFCT at 100 mg/kg (HG group) evidently enhanced the enzyme activities of GSH-Px in all tested tissues (*P* < 0.05 or *P* < 0.01). In addition, the MG (*P* < 0.05) and CG (*P* < 0.01) groups of CFCT only increased the enzyme activities of GSH-Px in blood.

## Discussion

*Cordyceps* is a potential source for the discovery of anticancer drugs. It has been well documented that *Cordycep*s-derived extracts and/or compounds showed potent inhibitory activities against different cancer cell lines *in vitro* through different mechanisms as reviewed previously [6]. However, *in vitro* methods are susceptible to false-positive and false-negative results [19]. Therefore, it is emphasized that antitumor efficacy testing in rodents should be used to predict the possible clinical response. Until now, only a few of *Cordyceps*-derived polysaccharides and/or water extracts have been investigated for their antitumor potentials in the tumor-bearing animal models [20–22], and *in vivo* antitumor effects of *Cordyceps-*derived lipophlic extracts and/or chemical entities have yet to be elucidated.

This study showed that CFCT, a lipophlic extract of *C. taii*, possessed moderate cytotoxicity against human cancer cell lines *in vitro*, and furthermore it could remarkably inhibit tumor growth in S180 tumor-bearing KM mice and melanoma B16F10-bearing C57BL/6 mice. CFCT also prolonged the survival time and increased the survival rate in both models. The histopathological results indicated that each administration group of CFCT could effectively inhibit the tumor growth and nuclear fission, and lead to necrosis of tumor tissue, especially for the HG and CG groups. It is known that both the innate and the adaptive immune systems are active against cancers [23]. Thymus is a top central immune organ, and spleen is one of the biggest peripheral immune organs [24, 25]. The viscera indices of thymus and spleen are recognized as the preliminary indicators to reflect the body’ immune function [15]. Previous studies have suggested that the crude extract of *Cordyceps* could inhibit the tumor growth associated with the elevated thymus and/or spleen index [26]. In this study, the thymus index of C57BL/6 mice and the spleen index of KM mice were increased when treated with CFCT of 100 mg/kg and 50 mg/kg, respectively (Table 1 and Table 2). The results imply that CFCT might have different immune regulation mechanisms on two mouse models. The increased thymus index suggested that CFCT could potentiate cell-mediated immunity of C57BL/6 mice, and the increased spleen index implied that CFCT could enhance humor-mediated immunity of KM mice. However, a bit confusing fact is that the spleen index of C57BL/6 mice and the thymus index of KM mice were slightly decreased under treatment with CFCT. Because the immune organ index is just a superficial indicator of immune function, the precise effects of CFCT on immune system need further investigation.

The presence of metastasis is the major cause of cancer mortality in millions of cancer patients, and it is urgently required to develop new anticancer agents with antimetastatic activities. Melanoma is one of the most aggressive skin cancers with a high metastatic potential, and it is difficult to be curbed [27]. In most cases, the lung is the first organ that tumor cells detaching from primary tumors encounter, making it a major site for tumor metastasis. In this study, the lung metastasis of melanoma B16F10 cells was effectively inhibited by CFCT alone and in combination with CTX, but its mechanism of action remained unclear. One group reported that the combination of chemotherapeutic agent methotrexate and water extract of *C. sinensis* (WECS) could inhibit the hematogenic lung metastasis in melanoma B16-BL6-bearing C57BL/6 J mice [28]. WECS could also reduce the hepatic metastasis of melanoma B16-F0 cells in C57BL/6Cr mice, and its mechanism of antimetastatic action was associated with reducing the hepatocyte growth factor, and accelerating the secretion of tissue inhibitor of metalloproteinase-1 [29, 30]. Another group demonstrated that exopolysaccharide of *C. sinensis* inhibited tumor growth and metastasis in the lungs and livers of B16 melanoma-bearing mice by reducing c-Myc, c-Fos, and vascular endothelial growth factor receptor (VEGF) expression levels [31]. Here VEGF is a central molecule involved in the angiogenesis and metastasis and regarded as a cancer therapeutic target of the angiogenesis and metastasis [32, 33]. Interestingly, Ruma et al. recently observed that *C. militaris* extract could remarkably suppress melanoma growth by down-regulating VEGF expression and inducing the cell apoptosis [21]. This work implies that the anticancer and antimetastatic mechanisms of CFCT are possibly associated with the anti-angiogenicity and apoptosis induction.

It is well known that oxidative stress is involved in a variety of pathological processes, such as cancer, neurodegenerative diseases, ageing, and cardiovascular diseases [34, 35]. The role of redox signaling in cancer progression is well accepted. In general, a higher level of reactive oxygen species (ROS) is essential for cancer cell survival, proliferation, and metastasis via the regulation of redox signaling. H_2_O_2_ can function as a signaling molecule in tumor angiogenesis by inhibiting tyrosine phosphatase activity in growth factor signaling transduction [36, 37]. Therefore, therapeutic strategies aimed at removal of free radicals, especially H_2_O_2_, might be a reasonable choice for the treatment and prevention of cancer. Recently many extracts and products of *Cordyceps* have been shown to have significant antioxidant properties [38]. Polysaccharides from *C. sinensis* and *C. militaris* possessed strong antioxidant activities such as scavenging free-radicals, inhibiting lipid peroxidation, chelating metal ion, etc. [39, 40]. Our previous studies demonstrated that polysaccharides of *C. jiangxiensis* and *C. taii* possessed antioxidant activities *in vitro* and *in vivo* [9, 41]. The facts suggested that *Cordyceps* may be a promising source of antioxidants or antioxidant compounds. In the case of B16F10-bearing mice treated with CFCT of 100 mg/kg, the activities of GSH-Px, one of primary antioxidant enzyme that converts glutathione to oxidized glutathione and metabolizes H_2_O_2_ and lipid hydroperoxides into harmless products, were found to be significantly increased in blood, brain and liver tissues compared to the model mice (Fig. 7). Our data indicate that the antitumor activity of CFCT could be due to, at least in part, its ability to enhance the GSH-Px activity of organisms. Therefore, the antioxidant properties of CFCT *in vitro* and *in vivo* and whether the tumor inhibition of CFCT is linked to antioxidation are to be addressed here.

Previous studies demonstrated that the antitumor effective components of water extracts in *C. sinensis* and *C. militaris* included cordycepin and polysaccharide [21, 31, 42]. Chemical compositions of lipophlic extracts in *Cordyceps* species are more complicated and diversified. The definite antitumor effect of CFCT *in vivo* provided a substantial foundation for the further development of new chemopreventive lead compounds from *C. taii*. Recently, a bioassay-guided separation of CFCT was conducted by combining silica gel column chromatography and semipreparative high-performance liquid chromatography with SRB assay by our group [43]. Although cordycepin, the most well-known anticancer substance in *Cordyceps*, was not detected in *C. taii* [44], three known compounds - helvolic acid, cytochalasin C and zygosporin D, and three new cytochalasin derivatives, were isolated and identified from CFCT [13, 43]. Furthermore, we found that cytochalasin C, zygosporin D and three new cytochalasin derivatives displayed remarkable cytotoxicity against both lung cancer cell lines 95-D and A-549 *in vitro*, while no cytotoxic effect to human normal lung cells MCR-5. These findings indicated that those five compounds had excellent selectivity between normal and cancer cells, and their molecular mechanism of anticancer deserved an in-depth study [43]. Helvolic acid, a fusidane skeleton triterpenic acid with antimicrobial activity, was identified from *Cordyceps* species for the first time, and was also verified that it had potent cytotoxic activity against various human cancer cell lines [13]. Through these researches, the concrete effective components of CFCT could be understood at least partially, which is important to our searching for cancer chemopreventive compounds from the medicinal mushroom *C. taii*.

## Conclusion

CFCT was found to have notable antitumor and antimetastatic activities *in vivo*. The possible mechanism of the anticancer effect of CFCT may be related to its anti-proliferation, antioxidant, and immunoregulatory effects, but a further investigation is required. To our knowledge, this is the first report on antitumor and antimetastatic activities by the lipophlic extract of *C. taii*.
