# Erratum to: ‘Antitumor and antimetastatic activities of chloroform extract of medicinal mushroom *Cordyceps taii* in mouse models’

**DOI:** 10.1186/s12906-015-0797-y

**Published:** 2015-08-18

**Authors:** Ru-Ming Liu, Xiao-Jie Zhang, Gui-You Liang, Yong-Fu Yang, Jian-Jiang Zhong, Jian-Hui Xiao

**Affiliations:** Guizhou Center for Translational Medicine & Laboratory of Cell Engineering, Affiliated Hospital of Zunyi Medical University, Zunyi, 563000 China; Department of Pathology, Affiliated Hospital of Zunyi Medical University, Zunyi, 563000 China; State Key Laboratory of Microbial Metabolism, Joint International Research Laboratory of Metabolic & Developmental Sciences, and School of Life Sciences & Biotechnology, Shanghai Jiao Tong University, Shanghai, 200240 China

Unfortunately, the original version of this article [1] contained an error. Two of the affiliations were incorrect. The correct affiliations can be found below.

Correspondence: jhxiao@yahoo.com; jjzhong@sjtu.edu.cn

^1^Guizhou Center for Translational Medicine & Laboratory of Cell Engineering, Affiliated Hospital of Zunyi Medical University, Zunyi 563000, China.

^3^State Key Laboratory of Microbial Metabolism, Joint International Research Laboratory of Metabolic & Developmental Sciences, and School of Life Sciences & Biotechnology, Shanghai Jiao Tong University, Shanghai 200240, China.

^†^Both authors contributed equally to this work.
